# Falling with Style: Bats Perform Complex Aerial Rotations by Adjusting Wing Inertia

**DOI:** 10.1371/journal.pbio.1002297

**Published:** 2015-11-16

**Authors:** Attila J. Bergou, Sharon M. Swartz, Hamid Vejdani, Daniel K. Riskin, Lauren Reimnitz, Gabriel Taubin, Kenneth S. Breuer

**Affiliations:** 1 School of Engineering, Brown University, Providence, Rhode Island, United States of America; 2 Department of Ecology and Evolutionary Biology, Brown University, Providence, Rhode Island, United States of America; University of Oxford, UNITED KINGDOM

## Abstract

The remarkable maneuverability of flying animals results from precise movements of their highly specialized wings. Bats have evolved an impressive capacity to control their flight, in large part due to their ability to modulate wing shape, area, and angle of attack through many independently controlled joints. Bat wings, however, also contain many bones and relatively large muscles, and thus the ratio of bats’ wing mass to their body mass is larger than it is for all other extant flyers. Although the inertia in bat wings would typically be associated with decreased aerial maneuverability, we show that bat maneuvers challenge this notion. We use a model-based tracking algorithm to measure the wing and body kinematics of bats performing complex aerial rotations. Using a minimal model of a bat with only six degrees of kinematic freedom, we show that bats can perform body rolls by selectively retracting one wing during the flapping cycle. We also show that this maneuver does not rely on aerodynamic forces, and furthermore that a fruit fly, with nearly massless wings, would not exhibit this effect. Similar results are shown for a pitching maneuver. Finally, we combine high-resolution kinematics of wing and body movements during landing and falling maneuvers with a 52-degree-of-freedom dynamical model of a bat to show that modulation of wing inertia plays the dominant role in reorienting the bat during landing and falling maneuvers, with minimal contribution from aerodynamic forces. Bats can, therefore, use their wings as multifunctional organs, capable of sophisticated aerodynamic and inertial dynamics not previously observed in other flying animals. This may also have implications for the control of aerial robotic vehicles.

## Abstract 2

How do bats turn in mid-air in order to land or recover from a fall? This study compares numerical simulation with high-speed video, demonstrating that bats are able to reorient solely through inertial forces by maneuvering their heavy wings.

## Author Summary

Bats demonstrate remarkable agility in flight, reorienting from a horizontal flying position to a heels-over-head roosting position and recovering from aerial stumbles with ease. In this paper, we demonstrate that bats are able to execute these elegant maneuvers using primarily inertial forces, by controlled articulation of their heavy wings. Video from multiple high-speed cameras is used to create a digital representation of several flights in which bats either land on the ceiling or attempt to land and then recover from the subsequent fall. We observe that even at low speeds, when aerodynamic forces are likely to be small, the bats are able to reorient using combinations of asymmetric wing motions. A “minimal model” of a bat is proposed, which consists of a body and wings able to execute simplified motions. The dynamics of this minimal bat are then simulated and we are able to show that both roll and pitching motions, similar to those observed in the videos, can be achieved using specific sequences of wing motions. To confirm the general predictions of the minimal model, we simulate a fully articulated wing and body model, using as input the complex wing articulations measured from a series of 12 flights. We compare the simulated body orientation with the measured body orientation, finding excellent agreement between the two and thus supporting the hypothesis that inertial forces and not aerodynamic forces are largely responsible for these low-speed aerial maneuvers.

## Introduction

The ability to effectively maneuver is integral to animal life in natural environments. Most animals must maneuver throughout their life cycles: capturing prey, escaping from predators, migrating, foraging, and mating often put stringent demands on motor systems [1,2]. Because of this, there is strong selective pressure to evolve effective locomotion. Flying animals, in particular, have evolved a wide array of maneuvering strategies [3–7], and while many studies have focused on insects and birds, the flight mechanisms employed by the third group of extant flyers—bats—have historically received far less attention and are less well understood [8].

The relative lack of attention paid to bat flight is surprising. Bats have evolved a particularly impressive capacity to control their flight and are regarded as highly maneuverable flyers for their size [9,10]. We suggest that manipulation of inertial dynamics may be an under-appreciated aspect of animal flight control that can be relevant for both flapping and gliding flight, and could figure importantly in the evolution of powered flight from directed aerial descent. Bats often maneuver through cluttered environments such as caves or forests [11], and perform highly acrobatic aerial maneuvers such as landing upside down [12]. Bats’ maneuverability derives, in part, from their capacity to modulate their wing shape using numerous independently controlled joints [12,13]. With growing interest in understanding the breadth of performance capacities of these animals, studies have begun to probe bats’ sophisticated ability to manipulate air flow around their wings [14,15].

The dominant roosting position of bats, hanging vertically head-under-heels, requires reorientation from the flying position, which is head-above-heels with the vertebral column parallel to the ground. Consequently, the bats must perform rapid, high-precision, aerial rotations to orient and attach themselves to their landing target. If they fail to grasp targets, they must perform similar aerial rotations to self-right. The need to routinely perform these extreme aerial maneuvers raises an important question: how do bats generate the necessary forces to reorient themselves without falling away from their intended landing targets, given that they are not able to hover upside down? A possible answer is that bats may reorient by differential modulation of the moment of inertia in moving apendages, thereby executing zero-angular-momentum turns.

Inertial reorientation plays an important role in the locomotion of numerous animals. Perhaps the most famous and well-studied example is falling cats, which reorient inertially to land on their feet by asymmetrically twisting their bodies [16–18]. Humans also control various aerial movements, including somersaults and dives, by varying our bodies’ moment of inertia (e.g., [19]). Numerous other taxa, including lizards, primates, and rodents, inertially maneuver and reorient by moving various appendages and body segments [20–22]. Inertial reorientation has also been shown to contribute to certain bird flight maneuvers [23,24].

Bat wings contain many bones and muscles, and consequently, bats possess the heaviest wings amongst extant flyers, normalized by body mass [25,26]. The mass of the wings, along with recent observations showing the coupling of trunk movement with wing motion [27], suggest that movement of bat wings may be an effective means of inducing inertial reorientation during a landing maneuver.

Here, we describe a series of experiments in which we elicited repeated landing and falling maneuvers from several bats. Using a model-based tracking algorithm [28], we reconstructed the complex wing kinematics of these maneuvering bats. Using a simple analytical model, we demonstrate that these kinematics are capable of producing acrobatic landing maneuvers. Finally, the kinematic data, along with a detailed treatment of the anatomical distribution of mass in the head, trunk, and wings, are used in a dynamical model that simulates the forces acting on the bat as it lands or falls. From these data and models, we determine the role of wing inertia during these maneuvers.

## Materials and Methods

### Bat Flight Measurements

**Fig 1:**
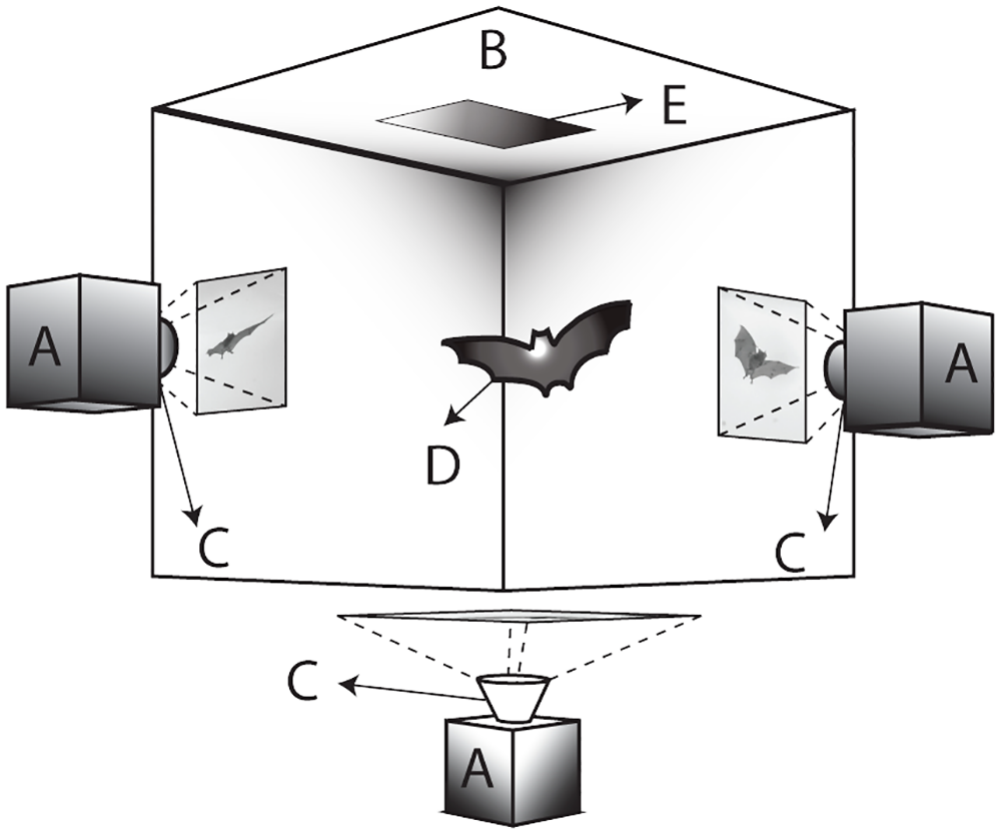
Apparatus for motion capture of landing bats. Videos are captured using three high-speed cameras (A) equipped with 50 mm lenses (C), at a frame rate of 1,000 frames per second. A uniform background (B) made of heavy-duty paper is placed behind the bat (D) to enhance contrast and visibility. The landing pad (E) is removed in order to generate a falling-and-recovery maneuver.

Two species of bats were used in our study: Seba’s short-tailed bats, *Carollia perspicillata* (three individuals), and Lesser dog-faced fruit bats, *Cynopterus brachyotis* (two individuals). All bats were housed either at the Concord Field Station of Harvard University (*C*. *brachyotis*) or at Brown University (*C*. *perspicillata*). We encouraged bat subjects to land repeatedly in a single location in view of three high-speed cameras by covering the ceiling and walls of a flight corridor (8.3 m × 1.0 m × 2.4 m) with smooth plastic sheets and heavy-duty paper, except for a small, square landing pad made out of a white mesh (Fig 1). Through training, bats learned to regularly land on the small area provided by the landing pad. To elicit “failed landing” maneuvers, we modified the landing experiment by removing the landing pad. Bats would attempt to grasp the missing landing pad and on failure would begin to fall, quickly reorient, and then fly out of the test area.

Three high-speed cameras (Photron PCI-1024 model 100k) equipped with 50 mm lenses were placed approximately orthogonally and used to record wing and body kinematics of maneuvering bats. All videos were recorded at 1,000 frames per second (fps), which allowed us to acquire between 500 and 1,500 frames per maneuver, or equivalently, between 125–175 frames per wingbeat. We lit the scene using diffuse white light. To further increase lighting and scene contrast, we covered the walls across from each camera with heavy-duty white paper.

The three-dimensional wing and body kinematics of maneuvering bats were reconstructed from high-speed video using the method described in [28]. Our model-based tracking system incorporates a Kalman filter and a priori biomechanical constraints to recover the complex motions associated with bat flight maneuvers. We use this method to extract six degrees of freedom describing a bat’s body position (*x*, *y*, and *z*) and orientation (*θ*, *ψ*, and *ϕ*), 23 joint angles describing the articulated pose of each of the bat’s wings, and 38 parameters describing the bat’s geometry (i.e., shoulder and hip positions, body dimensions, and bone lengths). In addition to recovering kinematics, the Kalman filter technique produces accurate estimates of both time derivatives and experimental uncertainties.

Using these methods, we elicited repeated landing maneuvers from three Seba’s short-tailed bats, *Carollia perspicillata*, and analyzed *n* = 9 flight sequences, each of which comprised four to five wingbeats. In addition, we performed video analysis on *n* = 2 flight sequences performed by Lesser dog-faced fruit bats, *Cynopterus brachyotis*, using video data originally recorded by Riskin et al. [29]. (Note: Riskin et al. [29] did not measure wing kinematics. Here, we have reanalyzed the raw video obtained for that study to quantify wing and body motion from two of those trials. Note that the definition of Euler angles used to specify the orientation of the bat differ between the current work and Riskin et al. [29].)

To determine the role inertial changes play in a bat’s control of flight maneuvers, we measured the distribution of mass of the bat’s wing and body as in [30]. The right wing of previously deceased bat specimens was dissected and each of 32 constituent fragments was weighed with a resolution of 0.001 g (see S1 and S2 Tables for a summary of the mass distribution).

### Model System Computations

To test the hypothesis that inertial changes due to wing movements can result in the reorientation of the bat’s body, we introduce two numerical models of a maneuvering bat. The first model is a highly simplified “minimal model” that captures the key kinematic properties for maneuvering. Our minimal bat comprises a rigid trunk and head with moment of inertia along the roll axis defined by *I*_*b*_. The minimal bat has two rectangular wings with equal masses, *M*_*w*_ (here, and elsewhere, subscript “*w*” denotes a property of the wing). For simplicity, the flapping axis of the wings is assumed to be the same as the body longitudinal axis of rotation. In addition to flapping up and down, each wing can be independently extended or withdrawn, indicated by the parameter *e*_*l*_ and *e*_*r*_ for the left and right wings, respectively. When each wing is fully extended (*e*_*r*_ = *e*_*l*_ = 1), they have equal wingspan, *s*, chord, *c*, and moment of inertia (taken about the wing joint), *I*_*w*_. The model also allows for each wing to be protracted or retracted, quantified by the angles *θ*_*r*_ and *θ*_*l*_. We restrict our investigation to wingbeats with a stroke plane perpendicular to the bat’s longitudinal axis and with left-right symmetric stroke angles *ϕ*_*l*_(*t*) = *ϕ*_*r*_(*t*) = *ϕ*_*w*_(*t*). The minimal model is implemented in MATLAB using standard simulations techniques.

In addition to the minimal model, we have also developed a “detailed dynamical model” that can mimic the full complexity of the observed kinematics and is capable of simulating full inertial dynamics. The model simulates a bat as an articulated rigid body in complete analog to the tracked maneuvering kinematics. We connect the 14 bones in each of the bat’s wings (S1 Fig) and model the system as follows: The bat’s head and torso are modeled as rigidly attached ellipsoids of uniform density (S1 Table) and with three translational and three rotational degrees of freedom (described by *x*−*y*−*z* and Euler angles roll, pitch, and yaw: *ψ*, *θ*, *ϕ*). The humerus is connected to the torso at the shoulder with a joint that has 3 rotational degrees of freedom. The forearm is connected to the end of the humerus with an elbow joint that has one rotational degree of freedom. The wrist is connected to the end of the forearm and modeled as a point mass. Both *C*. *perspicillata* and *C*. *brachyotis* have a short thumb that contains little mass. The second finger is fused to the third finger and is modeled as a single finger. The carpometacarpal joint connecting each finger to the wrist has three degrees of freedom. Each metacarpophalangeal and interphalangeal joint has a single degree of freedom. The femur is attached to the torso with a hip joint having three degrees of freedom. Lastly, the tibia is attached to the femur with a joint containing a single degree of freedom. We modeled all of the bones as thin rods of uniform density with mass determined from a dissection of a representative individual of each species (S2 Table). We treat the wing membrane skin as massless (S2 Table). In sum, we model a total of 52 degrees of freedom (six for the body + 23 for each wing).

To solve for the orientation changes associated with wing motions, we prescribe the wing kinematics (obtained from the measurements described above) and solve a recursive Newton-Euler algorithm, standard for simulating the motion of rigid body mechanics [32]. The dynamics of this model can be written as:

$$M(q)\ddot{q}+C(q,\dot{q})=\tau_{i}+\tau_{ext},$$ (1)

where *τ*_*i*_ is the vector of generalized torques exerted by the bat to adjust each wing joint, *τ*_*ext*_ is the vector of generalized external torques and forces (i.e., aerodynamics). In this work, we only consider inertial motions (*τ*_*ext*_ = 0). The generalized position vector *q* parametrizes the 52 degrees of freedom of the bat. **M**(*q*) and $C(q,\dot{q})$ are the system mass matrix and Coriolis matrix, respectively. These quantities are determined by the bat’s morphology and motion, as determined from the kinematic tracking (S2 and S1 Figs). The system of equations is solved in a custom-written Python code, using standard numerical techniques.

All experiments were conducted with the approval and under the oversight of the Brown University Institutional Animal Care and Use Committee (IACUC). Data and simulation codes are available from the Dryad Digital Repository, http://dx.doi.org/10.5061/dryad.21qs5 [31].

## Results and Discussion

**Fig 2:**
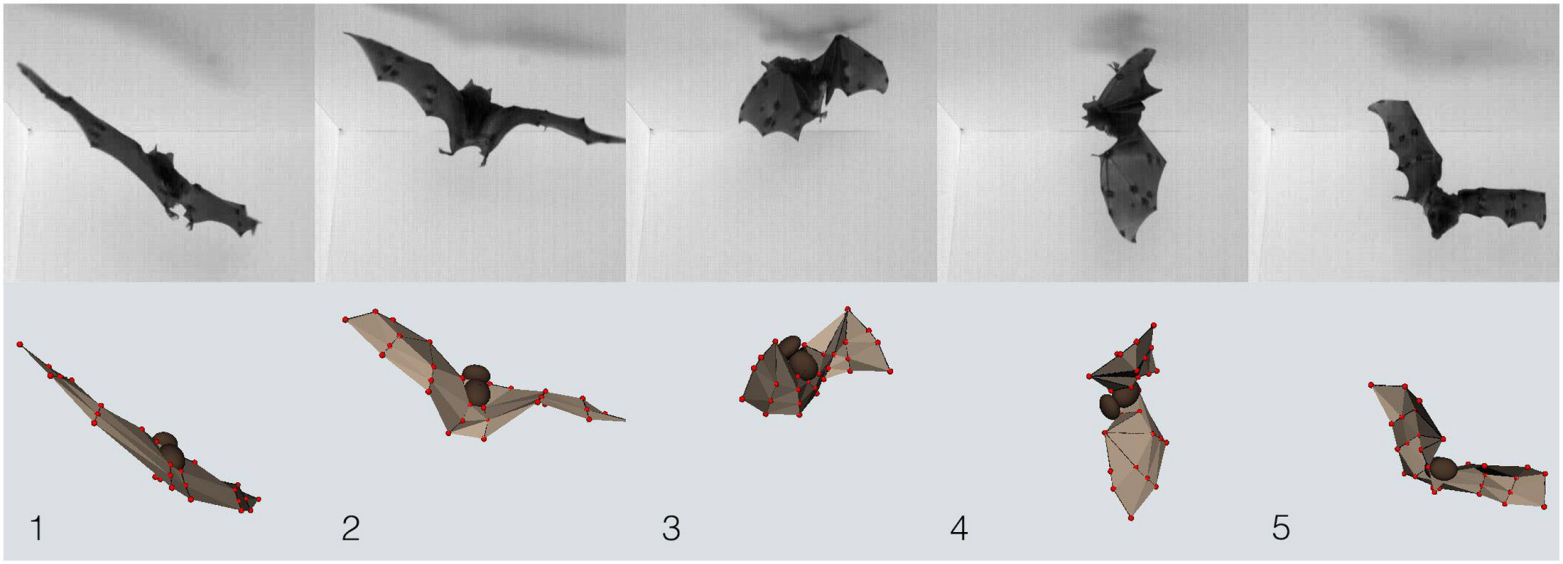
Bats rapidly reorient their bodies during landing. (Above) Selected images from high-speed recordings of *C*. *perspicillata* executing a landing maneuver and, upon failing to find a landing site, executing a righting maneuver. (Below) Corresponding 3-D reconstruction of the 52-degree-of-freedom flight kinematics. The images from left to right correspond to *t* = 0.185 s, 0.26 s, 0.335 s, 0.41 s, and 0.485 s (also, see Fig 3). To give a sense of scale, *C*. *perspicillata* have a characteristic tip-to-tip wingspan of approximately 30 cm. Tracked video data available in file tracked_data.zip from the Dryad Digital Repository, http://dx.doi.org/10.5061/dryad.21qs5 [31].

In Fig 2 we show a sequence of still images from a sample video (S1 Video) of a landing and subsequent righting maneuver performed by *C*. *perspicillata*. Immediately below each frame is the corresponding digital representation of the bat reconstructed from our digitizing procedure. Qualitatively, we found that both landing and righting maneuvers are highly stereotyped and that the wing and body kinematics of bats are quite similar across flight sequences for each species. This is consistent with a previous study of three bat species, including the two species of this study, that observed low within-species variation in both landing kinematics and ceiling impact forces generated during landing, although the two species differed in details of landing strategy [29]. In the following discussion, we analyze these maneuvers with focus on a single flight sequence performed by *C*. *perspicillata*. We then demonstrate that the mechanisms we identify can be used to explain each maneuver in our dataset.

**Fig 3:**
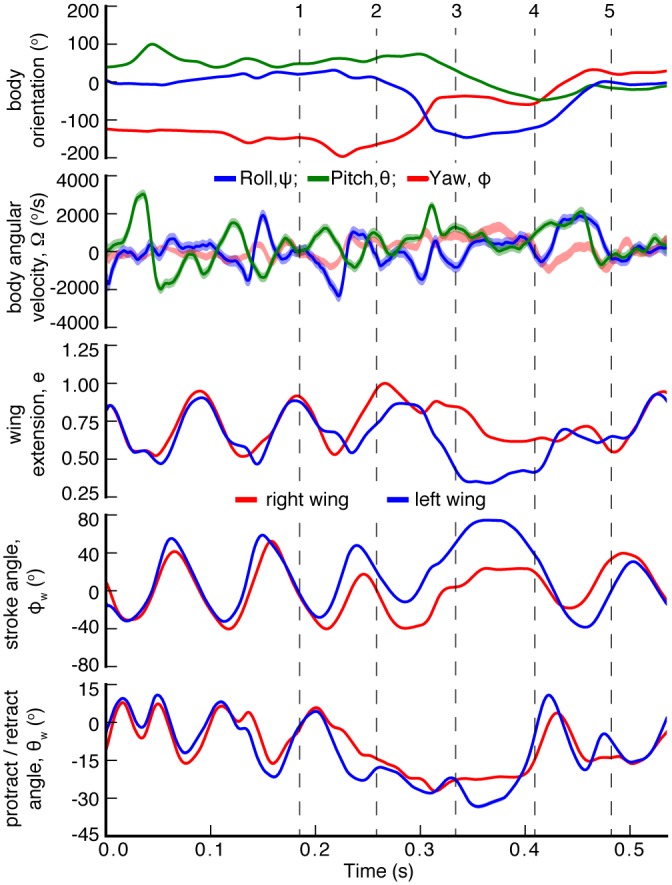
Changes to a landing bat’s body orientation, roll, *ψ*, pitch, *θ*, and yaw, *ϕ*, are prompted by pronounced changes in its wing kinematics. The top two frames indicate body orientation and angular velocity (about body-fixed axes). The lower three frames show simplified wing kinematics: instantaneous wing extension, *e*, wing stroke angle, *ϕ*_*w*_, and protraction-retraction angle, *θ*_*w*_. The departure from symmetric left-right wing motion coincides with changes in the body orientation. The five moments in time indicated by dashed vertical lines correspond to the images shown in Fig 2.

The kinematics of the body are represented by the *x*−, *y*−, and *z*− coordinates of the trunk and the three Euler angles: roll, *ψ*, pitch, *θ*, and yaw, *ϕ*, (Fig 3) [33]. The wing kinematics are fully described by the angles of each joint (S2 Fig), but can be conveniently parameterized using only three variables for each wing: the wing extension, *e*_*r*,*l*_, the ratio of the instantaneous to maximum span; stroke angle, *ϕ*_*r*,*l*_; and protraction-retraction angle, *ϕ*_*r*,*l*_ (Fig 3). The subscripts refer to the left and right wings, respectively. The reduced wing variables, *ϕ*_*l*_, *ϕ*_*r*_, *θ*_*l*_, and *θ*_*r*_ are distinct from the body Euler angles, *ϕ* and *θ*, which do not have subscripts. We employ this notation to maintain consistency with existing flight mechanics and flapping flight literature.

During the first 0.25 seconds of the flight sequence, the bat flies upwards towards the ceiling with steady orientation, *θ* ≈ 60°, *ψ* ≈ 0°, and *ϕ* ≈ −120°, and employs a left-right symmetric wingbeat (Fig 3). It then enters a period of maneuvering, *t* ≈ 0.25–0.5 sec., during which we observe changes along all three orientation axes, which are accompanied by substantial asymmetries in wing kinematics. During the landing portion of the flight sequence, 0.25 s<*t*<0.4 s, the bat transitions from a flying pose to a roosting pose, with its longitudinal axis pointed nearly vertical and its feet reaching into the air. During successful landings, the bat catches a landing target with its feet and comes to rest. When the bat fails to catch the landing target, as occurs in this particular sequence, the bat executes a righting maneuver and transitions back to a flying posture with the body axis oriented parallel to the ground.

During the portion of the flight sequence with the largest body reorientation, 0.25<*t*<0.45, the wing movements show substantial deviations from the simple symmetrical flapping characteristic of forward flight. First, the wings slow down, as evidenced by the reduced wingbeat frequency (Fig 3). As the body pitch changes, the bat’s wings are retracted during upstroke and protracted during downstroke (0.3 s<*t*<0.45 s). Finally, during the downstroke, at 0.25 s<*t*<0.30 s, the bat’s right wing is more extended than the left wing as body roll and yaw change.

### Minimal Model Simulations

Motivated by the kinematics observed during landings of *C*. *perspicillata*, we use our minimal model to first consider changes in a bat body’s roll angle, *ψ*, as a result of time-varying asymmetry in wing extension, *e*_*l*_ ≠ *e*_*r*_. For simplicity, and since we are interested in the dynamics of reorientation, we hold the position of the body constant in space. Furthermore, we require the yaw angle to remain constant. Although these constraints are somewhat restrictive, they have the advantage that the body dynamics simplify greatly and provide valuable physical insight. The Lagrangian, L, that represents the system dynamics can be written as:

$$L=\frac{1}{2}I_{p}{\dot{\theta }}^{2}+\frac{1}{2}I_{b}{\dot{\psi }}^{2}+\frac{1}{2}e_{r}^{2}I_{w}{\left({\dot{\phi }}_{w}+\dot{\psi }\right)}^{2}+\frac{1}{2}e_{l}^{2}I_{w}{\left({\dot{\phi }}_{w}-\dot{\psi }\right)}^{2}-V\;,$$ (2)

where the parameters are all as described earlier, with the addition of *I*_*p*_, which represents the moment of inertia in the pitching axis. The five terms in the equation correspond, respectively, to the kinetic energy of the body due to pitch and roll, the kinetic energy of the right and left wings, and the gravitational potential energy of the wing-body system. The equation of motion for the roll angle, *ψ*, follows directly from the Lagrangian, L, and is given by:

$$\frac{d}{dt}\left(\dot{\psi }+(e_{l}^{2}+e_{r}^{2})I^{*}\dot{\psi }+\left(e_{r}^{2}-e_{l}^{2}\right)I^{*}{\dot{\phi }}_{w}\right)=\tau_{e}-\frac{1}{I_{b}}\frac{\partial V}{\partial \psi },$$ (3)

where I* = *I*_*w*_ / *I*_*b*_, and *τ*_*e*_ is the external (aerodynamic) torque acting on the bat, normalized by the body moment of inertia, *I*_*b*_. Note that the equation does not depend on the pitch angle and that the last term in Eq 3, representing the torque due to gravity, is very small and contributes negligibly to roll dynamics.

To obtain an approximate estimate of the effect of aerodynamic forces, *τ*_*e*_, during this maneuver, we use a simple quasi-steady drag model (e.g., [34]). For this case, the aerodynamic force, *F*_*e*_, on each wing is approximated as:

$$F_{e}=\frac{4C_{d}}{3}\cdot \frac{1}{2}\rho_{f}v^{2}\bar{c}\bar{s},$$ (4)

where *C*_*d*_ is the drag coefficient of the wing, *ρ*_*f*_ is the fluid density, $\bar{c}$ is the average chord length of the wing, $\bar{s}$ is the base to tip span of the bat wing, and *v* is the instantaneous velocity of the wing’s centroid. The factor of 4/3 arises from the analysis and is necessary to account for the fact that the wing tip moves faster than the centroid and contributes more to the aerodynamic drag. Following [34], the effect of the external torque, *τ*_*e*_, can then be explicitly written as:

$$\tau_{e}=-C^{*}\left((\dot{\psi }-{\dot{\phi }}_{w})|\dot{\psi }-{\dot{\phi }}_{w}|e_{l}^{4}+(\dot{\psi }+{\dot{\phi }}_{w})|\dot{\psi }+{\dot{\phi }}_{w}|e_{r}^{4}\right),$$ (5)

where $C^{*}\equiv (\rho_{f}C_{d}\bar{c}{\bar{s}}^{4})/(8I_{b})$ is the scaled drag coefficient.

We solve Eqs 3–5 for the roll angle, *ψ*(*t*), subject to idealized kinematics *ϕ*_*w*_(*t*), *e*_*r*_(*t*) and *e*_*l*_(*t*), prescribed so that the minimal bat fully extends its wings at mid-downstroke and asymmetrically retracts its right wing during the upstroke. We use a representative flapping amplitude of ±30°:

$$\begin{array}{l} \phi_{w}(t)=30{}^{\circ}\text{cos}\left(2\pi t\right)\;,\\ e_{l}(t)=1\;,\\ \text{and }e_{r}(t)=\frac{1}{2}\left(1+\text{sin}(2\pi t)\right)\;. \end{array}$$ (6)

**Fig 4:**
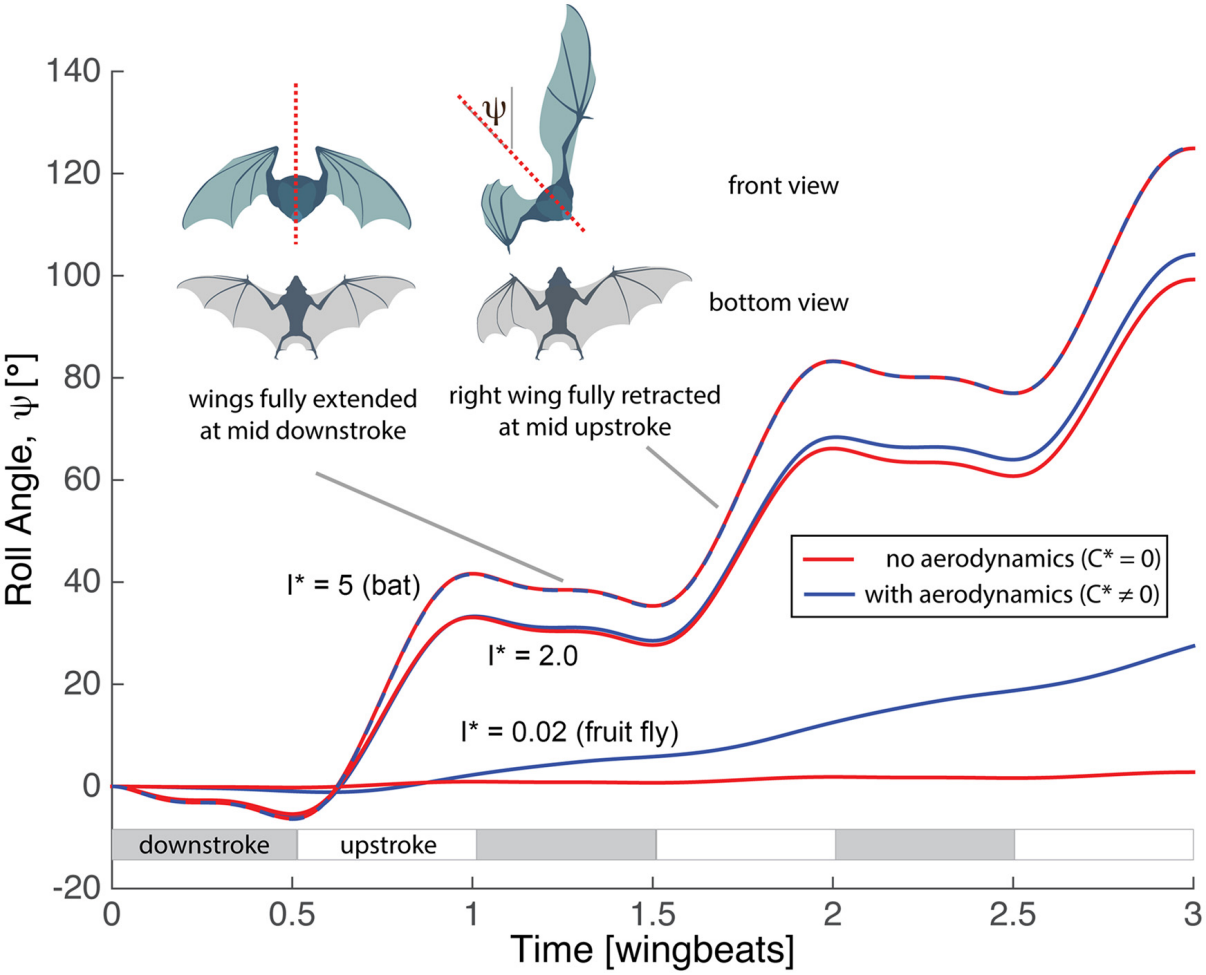
Minimal model of bat dynamics applied to body roll maneuver. Both wings are fully extended (*e*_*r*_ = *e*_*l*_ = 1) at mid-dowstroke, while one wing is fully retracted (*e*_*r*_ = 0) at mid-upstroke. For morphological parameters matched to those of *C*. *perspicillata* (I* = 5), simulations show that this asymmetric wing extension induces body roll and that aerodynamic forces do not influence the motion significantly. The response is insensitive to modest changes in the relative wing inertia (I* = 2), although when the morphological parameters are matched to those of fruit flies (I* = 0.02,C* = 0.05), aerodynamic forces dominate, while inertial forces have minimal effect on the body orientation. MATLAB code available in file minimal_simulation.zip from the Dryad Digital Repository, http://dx.doi.org/10.5061/dryad.21qs5 [31].

Using parameters matched to *C*. *perspicillata*: I* = 5 and C* = 1 (see Table 1), the simulated flights result in a net change in roll angle of approximately 40° with each wingbeat (Fig 4). If the aerodynamic forces are turned off (C* = 0, *τ*_*e*_ = 0), we see no change in the flight behavior, confirming that the rolling motion results from the asymmetric internal torques the bat applies as it flaps its heavy wings. When both wings are equally extended, the bat exerts a positive torque along its roll axis to flap its right wing and a negative torque to flap its left wing. As the right wing is retracted while the left wing remains extended, less torque is required to flap the right wing and the reaction torque on the body results in a net roll of the bat’s body. Parametric variations using the minimal model indicate that this behavior is preserved as long as the relative wing inertia remains significant. For example, a simulation using a substantially (2.5×) lower value of the wing inertia parameter, I* = 2, demonstrates that the model animal retains the ability to execute roll maneuvers using inertial dynamics (Fig 4), although a smaller roll angle is achieved and the relative importance of aerodynamic forces increases.

**Table 1:** Morphological and other constants used to simulate simplified dynamics using the minimal model.

Constant	Description	Bat	Fruit Fly [6]
*I*_*b*_	Roll Moment of Inertia	20 g cm^2^	1 × 10^−6^ g cm^2^
*I*_*w*_	Extended Wing Moment of Inertia (taken about the wing root)	100 g cm^2^	2 × 10^−8^ g cm^2^
I*	Ratio of Wing to Body Moment of Inertia	5	0.02
*ρ*_*f*_	Density of Air	1.2 × 10^−3^ g cm^−3^	1.2 × 10^−3^ g cm^−3^
*C*_*d*_	Drag Coefficient	2	2
$\bar{c}$	Mean Chord Length	5 cm	0.1 cm
$\bar{s}$	Mean Wingspan	11 cm	0.2 cm
C*	Dimensionless Aerodynamic Constant	1.0	0.05

However, the model predicts strikingly different turning behavior as the importance of wing inertia declines. Unlike heavy-winged bats, insects have relatively far lighter wings, and fruit flies have wings that are, relative to their body mass, nearly massless[6]. We repeat the simulations of Eq 3, this time matching morphological parameters to those of a fruit fly: I* = 0.02 and C* = 0.05 (Table 1) [6]. In addition, we perform the simulation without aerodynamics (C* = 0). Unlike the bat case, the fly maneuver is executed completely due to aerodynamic forces with no appreciable inertial contribution (Fig 4). The effectiveness of the rolling maneuver can be modified by changing kinematic parameters. For example, increasing flapping amplitude leads to a corresponding increase in the roll-per-wingbeat angle. Similarly, incomplete wing retraction during the upstroke reduces the roll angle achieved (see S4 Fig).

**Fig 5:**
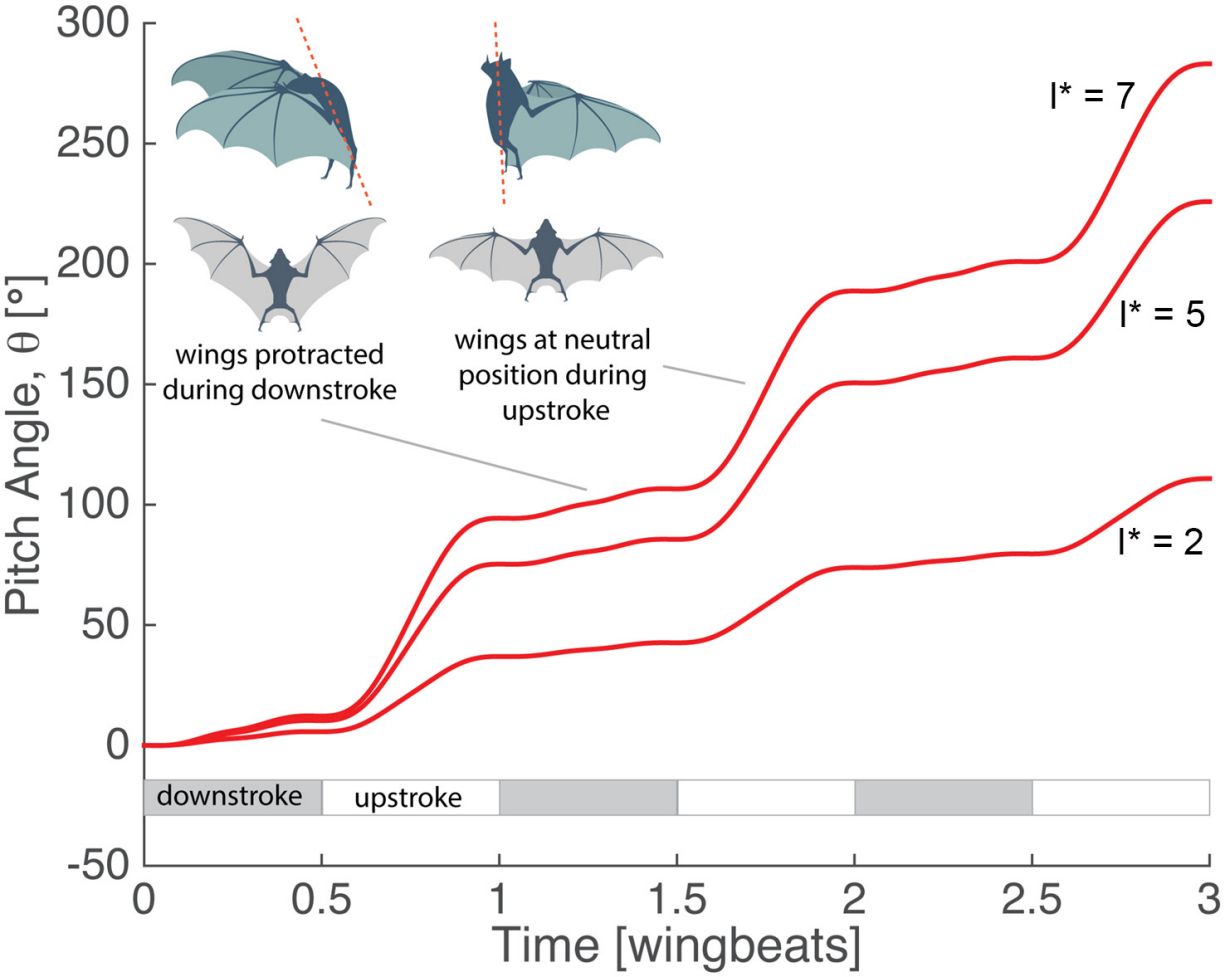
Minimal model of inertial mechanism bats use to adjust body pitch along with schematic of wing positions. The wings are protracted during the downstroke and retracted to the pitch-neutral position during the upstroke. Three values of the relative wing inertial parameter, I* = 2,5,7, are shown. I* = 5 corresponds to the morphology of *C*. *perspicillata*. MATLAB code available in file minimal_simulation.zip from the Dryad Digital Repository, http://dx.doi.org/10.5061/dryad.21qs5 [31].

Different combinations of asymmetric wing motions can be used to generate other inertially dominated reorientations. For example, to generate a net change in pitch angle, *θ*, the bat can protract and retract its wings asymmetrically with respect to the upstroke and downstroke (Fig 5).

Allowing for this protraction/retraction, a new Lagrangian can be defined:

$$\begin{array}{l} L=\frac{1}{2}I_{p}{\dot{\theta }}^{2}+\frac{1}{2}I_{b}{\dot{\psi }}^{2}+\frac{1}{3}M_{w}{\bar{s}}^{2}{\left({\dot{\phi }}_{w}-\dot{\theta }\text{sin}(\theta_{r})\right)}^{2}+\\ \frac{1}{12}M_{w}\left(4{\bar{s}}^{2}+{\bar{c}}^{2}\right){\left(\dot{\theta }\text{cos}(\theta_{r})\text{sin}(\phi_{w})+{\dot{\theta }}_{r}\text{cos}(\phi_{w})\right)}^{2}+\\ \frac{1}{12}M_{w}{\bar{c}}^{2}{\left(\dot{\theta }\text{cos}(\theta_{r})\text{cos}(\phi_{w})-{\dot{\theta }}_{r}\text{sin}(\phi_{w})\right)}^{2}\;-V. \end{array}$$ (7)

We again simulate a bat flapping with a ±30° wingbeat amplitude. During successive up- and downstrokes we prescribe left-right symmetric protraction of 22.5°, employing the first two terms of a Fourier-series expansion of a square wave function:

$$\begin{array}{l} \phi_{w}(t)=30{}^{\circ}\text{cos}\left(2\pi t\right)\;,\\ \theta_{l}(t)=\theta_{r}(t)=22.5{}^{\circ}\left(\frac{1}{2}+\frac{2}{\pi }\left(\text{sin}(2\pi t)+\frac{1}{3}\text{sin}(6\pi t)\right)\right)\;. \end{array}$$ (8)

This asymmetric wing motion results in a change in the body pitch angle of approximately 60° with each wingbeat (Fig 5). During the downstroke, the wings are swept forward and the body pitches up gently in reaction to the wing motion. As the wings start the upstroke, they are retracted to their neutral position, resulting in a more dramatic pitching response by the body. Although the overall trends are preserved over a range of values of the wing inertia parameter, I* (Fig 5), these dynamics are quite sensitive to the specifics of the sweep motion. Changing the amplitude of either the sweep motion, *θ*_*r*_,*θ*_*l*_, or the main flapping motion, *ϕ*_*w*_, can result in very different pitching dynamics and prescribing purely sinusoidal sweep instead of the first and third harmonic (Eq 8) leads to a less dramatic pitching motion. (see S5 Fig). Furthermore, the introduction of aerodynamic forces (C* ≠ 0) to the simulation also affects the minimal model results. This indicates that both inertial and fluid forces can be marshaled successfully to execute these maneuvers but that the complete motion must also incorporate subtle control of these two forces, likely mediated by the bats’ sophisticated sensory systems (e.g., [35,36]).

### Fully Articulated Model Simulations

If wing inertia can induce reorientations in the minimal bat, what is the contribution of wing inertia to the aerial rotations that we observe during flight trials? To answer this question, we use the fully articulated model of the bat to simulate the change in body posture in response to prescribed wing motions and in the absence of external (aerodynamic) forces.

**Fig 6:**
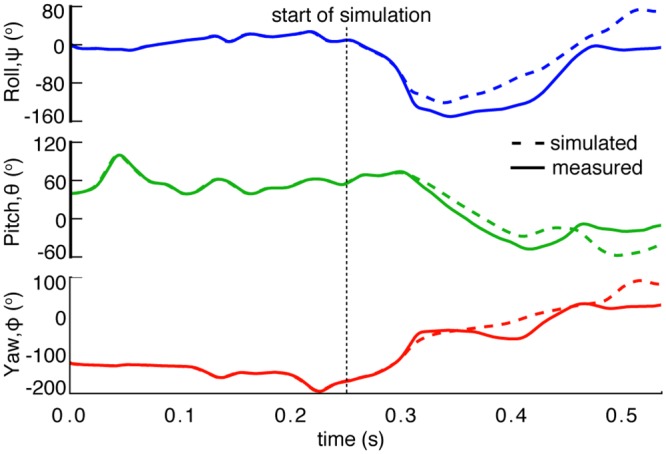
Inertial changes due to wing movement are sufficient to explain the complex reorientation of the bat’s body. Beginning at *t* = 0.25 sec, we simulate the free motion of a virtual bat due to the inertial effects of the measured wing motion. The simulated posture (dotted line) is compared with the measured posture (solid line). Data available in file EulerAngles.txt from the Dryad Digital Repository, http://dx.doi.org/10.5061/dryad.21qs5 [31].

We prescribe all 46 degrees of freedom of the two wings, as measured from the flight trials, and use standard robotic simulation techniques [32] to compute the change in the body’s orientation in response to these wing motions. We begin the simulation at the initiation of the landing maneuver and continue the simulation to the end of the tracked flight sequence. Unlike the minimal model simulations, these calculations do not represent cyclic wing motion, but rather complete simulations of the experimentally observed motion. For the attempted landing shown earlier (Fig 2) the simulation successfully recovers the complex aerial rotation and compares very well with the measurements, supporting our hypothesis that aerodynamic forces play little role in the maneuver and that, as a consequence, the motion is largely inertial (Fig 6). This assertion is supported by the observation that the center of mass of the bat follows a ballistic trajectory with its horizontal position moving at constant velocity and its vertical position in free fall. We find that, as long as the simulation is initiated after the start of the landing maneuver, the quality of the comparison is insensitive to the exact time of initiation. However, initiating the simulation prior to this point leads to increasing discrepancies between the simulation and the measurements, presumably due to the importance of aerodynamic forces during this portion of the maneuver.

**Fig 7:**
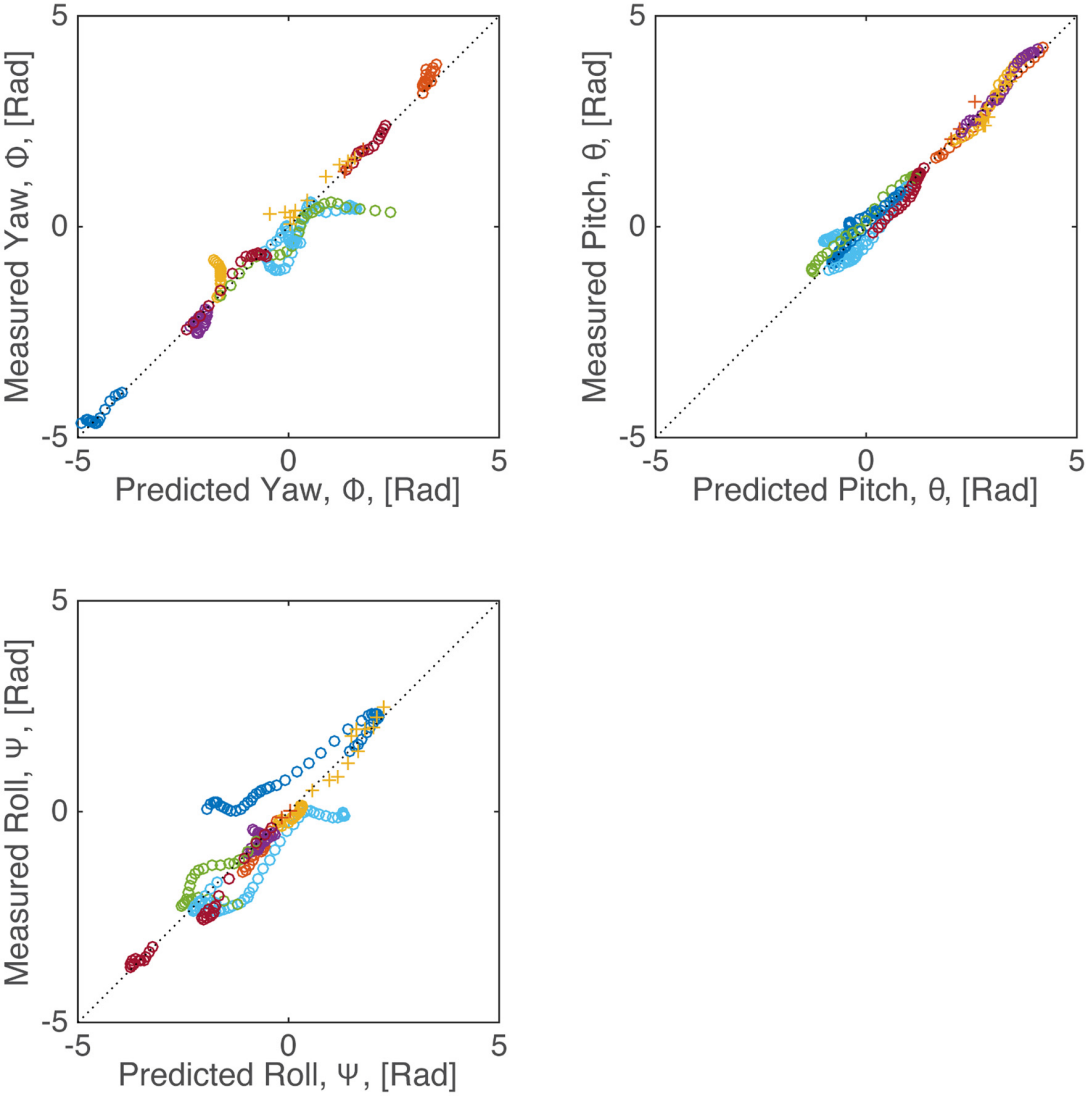
Comparison between the simulated and measured Euler angles for 11 flight sequences. The symbols denote the species (○: *C*. *perspicillata*; +: *C*. *brachyotis*); the colors identify each flight sequence. For clarity, every fifth data point during each flight sequence is plotted. For all flights, after subtracting the angle at *t* = 0 so as to remove bias, the correlations between the measured and predicted roll, pitch, and yaw angles is *R* ^2^ = 0.687, 0.935, and 0.721, respectively. Data available in file EulerAngles.txt from the Dryad Digital Repository, http://dx.doi.org/10.5061/dryad.21qs5 [31].

We repeat this detailed analysis for ten additional flight sequences, in each case using the measured wing kinematics as input to the simulation and solving the full dynamical equations (Eq 1) for the body motion. The bat’s body orientation predicted by the inertial simulation compares extremely well to measured body orientation (Fig 7) and strongly supports our hypothesis that wing inertia plays a dominant role in reorienting bats during landing and falling.

## Conclusions

How do bats land? Since almost all species of bats roost upside down, head-under-heels, a bat must perform a complex aerial rotation from a flying posture to a roosting posture. Similar maneuvers are required if the bat misses its landing mark and has to right itself, as well as during takeoff, when the bat drops from its roosting position and transitions to a flying posture. Using aerodynamic forces to achieve such body rotations is challenging; landing and takeoff necessarily take place at low flight speed, when the dynamic pressure is low. Hence, generating significant aerodynamic torques requires large wing extension (to attain sufficient wing area) and large amplitude wing motion, both of which are antagonistic to the confined space available in close proximity to the landing site.

Although bats surely employ some aerodynamic forces, even at low speeds, we show here that the bats in our investigation make extensive use of inertial forces, generated by folding and moving their relatively heavy wings, to reorient themselves during landing and falling. Since virtually all bats face the challenge of achieving major changes in body orientation during landing maneuvers [29], and they all share relatively massive articulated handwings [30], we suggest that such inertial maneuvering may be common among bats. The widespread use of these dynamics among many lineages [20–22] further suggests that the capacity to effectively manipulate limb and body segments for dynamic locomotor control may have evolved early in vertebrate history, or it may have evolved independently multiple times. Indeed, this ability may be of particular importance during leaping, gliding, and directed aerial descent (i.e., [37]), and fuller appreciation of inertial dynamics as a means of flight control may provide insight into potential evolutionary pathways to powered flapping flight. With this study, we illustrate the critical importance of this mechanism to extant bats. Here we present kinematic and morphological data from only two species, but we propose that the effect is likely quite general, given the similarities in the anatomical structure and the distribution of mass among diverse bat species [38,39]. This work opens the possibility that additional insight into the evolution of flapping and gliding flight could be gained by better understanding of inertial dynamics in mammalian gliders, whose complex takeoff and landing patterns may well employ a similar combination of aerodynamic and inertial effects [40].

The ability of our full inertial simulations to closely track measured body orientation demonstrates the dominance of inertial forces during these maneuvers. However, it is our minimal model that provides the greatest explanatory power. By abstracting the kinematics employed during pitch and roll maneuvers and by playing them through our idealized dynamical model, we are able both to elucidate how these motions are achieved and to assess the relative role of wing inertia and aerodynamics forces. Nevertheless, the model has specific limits. Although its ability to mimic inertial maneuvers at low speed, when aerodynamic forces can be neglected, is persuasive, it remains illustrative and does not predict dynamics with highly resolved detail. In particular, for the current analysis, translation and yaw motion were suppressed. This has the advantage that it decouples the pitch and roll motions but is nevertheless somewhat restrictive, and a more extensive, less constrained model would be interesting to explore. More importantly, the model’s representation of aerodynamic forces is currently crude; the sensitivity of the minimal model simulations to the value of C* exposes the limitations of this simple aerodynamic model, which does not incorporate spanwise variations, the local angle of attack, wing camber, etc. Nevertheless, the key result is robust: heavy-winged bats are capable of executing significant maneuvers at low speeds.

The ability to execute reorientation by inertial repositioning of wings also has implications for control of slow flight. Inertial reorientation confers additional control parameters to flyers with sufficiently heavy wings. Our results suggest that human-engineered flappers may benefit from employing an inertial control strategy [22]. Lastly, although heavy wings might be seen as a disadvantage for flight, due to the high inertial cost of flapping and the resistance that massive appendages present to rapid accelerations, these results suggest that, counterintuitively, heavy wings may be an asset instead of a liability for highly maneuverable flight at low speed.

## Supporting Information

S1 Fig The location of tracked markers, joints and degrees of freedom of the articulated bat model used for motion tracking and for the full dynamic simulation. Tracked markers are shown in blue. Each of the modeled joints is labeled on the left side of the figure. The head and trunk of the bat is modeled as a rigid body, and each wing is modeled as 13 rigid bones. Segments are modeled as connected using either ball-and-socket joints with three degrees of freedom, or as uniaxial hinge joints with a single degree of freedom, as indicated on the right side of the figure. The thumb and second finger are not modeled. The carpometacarpal joints are modeled as ball-and-socket joints, one for each of the fingers (3, 4, and 5). Each of the two most distal phalanges of fingers 3, 4, and 5 are modeled as a single bone.
(TIF)

S2 Fig Full 52-degree-of-freedom kinematics of bat during the failed landing and recovery maneuver. The sequence is shown in Figs 2 and 3 and the joints are as labeled in S1 Fig. Angles are shown in degrees. Kinematic parameters for the right wing are shown in red and the left wing in blue. For multi-axis joints, each degree of freedom is labeled with its corresponding Euler angle. Error bars, estimated by the tracking software, are indicated by the width of the lines.
(TIF)

S3 Fig Locations of the contributions to the mass distribution from the wing and body of a bat. The body was dissected into head and trunk; each wing was divided into 14 bones (B1-B14) and 18 membrane segments (M1–M18), each which were then individually weighed (see S1 and S2 Tables).
(TIF)

S4 Fig Sensitivity of rolling dynamics to parameter variations. The minimal model is used to simulate changes in roll angle, Ψ, due to asymmetric wing extension. Unless otherwise stated, the kinematic parameter are as defined by Eq 6. A: The flapping amplitude, *ϕ*_*w*_ is varied from ±20 to ±50 degrees. B: The degree to which the right wing folds during the upstroke is varied from 25% to 100%.
(TIF)

S5 FigSensitivity of pitching dynamics to parameter variations.The minimal model is used to simulate changes in pitch angle, *θ*, due to wing protraction during the upstroke. Unless otherwise stated, the kinematic parameter are as defined by Eq 8. A: The flapping amplitude, *ϕ*_*w*_ is varied from ±20 to ±50 degrees. B: The amplitude of the sweep (wing protraction) is varied from 10 to 40 degrees; C: The aerodynamic parameter, C* is varied from 0 to 1; D: The protraction waveform is varied smoothly from pure sinusoidal to the case discussed in Eq 8. This is achieved by re-defining the wing protraction as $\theta_{l}(t)=\theta_{r}(t)=22.5{}^{\circ}\left(\frac{1}{2}+\frac{2}{\pi }\left(\text{sin}(2\pi t)+\frac{A_{3}}{3}\text{sin}(6\pi t)\right)\right)$ and by varying *A*_*3*_ between 0 and 1.(TIF)

S1 Table Masses and dimensions of the head, trunk, and wings of bats. Values for wings are mean of left and right, which were within 0.1 g. For *C*. *brachyotis* the head and trunk were not individually weighed. Dimensions are given as length (longitudinal) × width × thickness.
(XLS)

S2 Table Mass distribution of the wing as a percentage of the total body mass for *C*. *brachyotis* and *C*. *perspicillata*. Segments labeled in S3 Fig.
(XLS)

S1 Video Still image from supplementary video. The video shows *Carollia perspicillata* attempting a landing and, on failing to grasp the ceiling, recovering from the fall. The left half of the frame shows the raw video while the right half shows the digital representation of the motion, reconstructed from the digitized kinematics.
(MP4)

## References

[1] Dickinson MH, Farley CT, Full RJ, Koehl MAR, Kram R, Lehman S. How animals move: An integrative view, Science 2000; 288, 100–106. 10753108 10.1126/science.288.5463.100

[2] Nathan R, Getz WM, Revilla E, Holyoak M, Kadmon R, Saltz D, et al A movement ecology paradigm for unifying organismal movement research, Proc. Natl. Acad. Sci. 2008; 105, 19052–19059. 10.1073/pnas.0800375105 19060196 PMC2614714

[3] Fry S, Sayaman R, Dickinson MH. The aerodynamics of hovering flight in *Drosophila* , J. Exp. Bio. 2005; 208, 2303–2318. 15939772 10.1242/jeb.01612

[4] Spedding G, Hedenström A, McArthur J, Rosen M. The implications of low-speed fixed-wing aerofoil measurements on the analysis and performance of flapping bird wings, J. Exp. Bio. 2008; 211, 215–223. 18165249 10.1242/jeb.007823

[5] Hedrick TL, Cheng B, Deng X. Wingbeat time and the scaling of passive rotational damping in flapping flight, Science 2009; 324, 252–255. 10.1126/science.1168431 19359586

[6] Bergou AJ, Ristroph L, Guckenheimer J, Cohen I, Wang ZJ. Fruit flies modulate passive wing pitching to generate in-flight turns, Phys. Rev. Lett. 2010; 104, 148101 20481964 10.1103/PhysRevLett.104.148101

[7] Lentink D, Muijres FT, Donker-Duyvis FJ, Leeuwen L. Vortex-wake interactions of a flapping foil that models animal swimming and flight, J. Exp. Bio. 2008; 211, 267–273. 18165254 10.1242/jeb.006155

[8] Norberg U. Vertebrate Flight: Mechanics, Physiology, Morphology, Ecology, and Evolution. Springer-Verlag 1990.

[9] Iriarte-Díaz J, Swartz SM. Kinematics of slow turn maneuvering in the fruit bat *Cynopterus brachyotis* , J. Exp. Bio. 2008; 211, 3478–3489. 18931320 10.1242/jeb.017590

[10] Hedenström A, Johansson LC. Bat flight: aerodynamics, kinematics and flight morphology, J. Exp. Bio. 2015; 218, 653–663. 25740899 10.1242/jeb.031203

[11] Norberg R. The pterostigma of insect wings an inertial regulator of wing pitch, J. Comp. Physiol. 1972; 81, 9–22.

[12] Hill JE, Smith JD. Bats: A Natural History. University of Texas 1992.

[13] Riskin DK, Willis DJ, Hedrick TL, Iriarte-Díaz J, Kostandov M, Chen J, et al Quantifying the complexity of bat wing kinematics, J. Theor. Bio. 2008; 254, 604–615. 18621062 10.1016/j.jtbi.2008.06.011

[14] Muijres FT, Johansson LC, Barfield R, Wolf M, Spedding GR, Hedenström A. Leading-edge vortex improves lift in slow-flying bats, Science 2008; 319, 1250–1253. 10.1126/science.1153019 18309085

[15] Hubel TY, Hristov NI, Swartz SM, Breuer KS. Time-resolved wake structure and kinematics of bat flight, Exp. Fluids 2009; 46, 933–943.

[16] Marey M. Des mouvements que certains animaux executent pour retomber sur leurs pieds, lorsqu’ils sont precipites d’un lieu eleve, Acad. Sci. 1894; 119, 714–717.

[17] Kane T, Scher M. A dynamical explanation of the falling cat phenomenon, International J. Solids and Structures 1969; 5, 663–666.

[18] Arabyan A, Tsai D. A distributed control model for the air-righting reflex of a cat, Biological Cybernetics 1998; 79, 393–401. 9851020 10.1007/s004220050488

[19] Zatsiorsky VM. Kinematics of human motion. Champaign, Illinois: Human Kinetics 1998.

[20] Dunbar DC. Aerial maneuvers of leaping lemurs: The physics of whole-body rotations while airborne, Amer. J. Primatology 1988; 16, 291–303. 10.1002/ajp.1350160402 32079371

[21] Essner RL. Three-dimensional launch kinematics in leaping, parachuting and gliding squirrels, J. Exp. Bio. 2002; 205, 2469–2477. 12124370 10.1242/jeb.205.16.2469

[22] Libby T, Moore TY, Chang-Siu E, Li D, Cohen DJ, Jusufi A, Full RJ. Tail-assisted pitch control in lizards, robots and dinosaurs, Nature 2012; 481, 181–184. 10.1038/nature10710 22217942

[23] Hedrick TL, Usherwood JR, Biewener AA. Low speed maneuvering flight of the rose-breasted cockatoo (*Eolophus roseicapillus*). ii. inertial and aerodynamic reorientation, J. Exp. Bio. 2007; 210, 1912–1924. 17515417 10.1242/jeb.002063

[24] Ros IG, Badger MA, Pierson AN, Bassman LC, Biewener AA. Pigeons produce aerodynamic torques through changes in wing trajectory during low speed aerial turns, J. Exp. Bio. 2015; 218, 480–490. 25452503 10.1242/jeb.104141

[25] Ellington CP. The aerodynamics of hovering insect flight. I. The quasi-steady analysis, Phil. Trans. R. Soc. B 1984; 305, 1–15.

[26] van den Berg C, Rayner J. The moment of inertia of bird wings and the inertial power requirement for flapping flight, J. Exp. Bio. 1995; 198, 1655–64. 9319563 10.1242/jeb.198.8.1655

[27] Iriarte-Díaz J, Riskin DK, Willis DJ, Breuer KS, Swartz SM. Whole-body kinematics of a fruit bat reveal the influence of wing inertia on body accelerations, J. Exp. Bio. 2011; 214, 1546–1553. 21490262 10.1242/jeb.037804

[28] Bergou AJ, Swartz S, Breuer K, Taubin G. 3D reconstruction of bat flight kinematics from sparse multiple views, in *Computer Vision Workshops (ICCV Workshops)*, *2011 IEEE International Conference on Computer Vision Theory and Applications*. pp. 1618–1625. IEEE.

[29] Riskin DK, Bahlman J, Hubel TY, Ratcliffe JM, Kunz TH, Swartz SM. Bats go head-under-heels: the biomechanics of landing on a ceiling, J. Exp. Bio. 2009; 212, 945–953. 19282491 10.1242/jeb.026161

[30] Riskin DK, Bergou A, Breuer KS, Swartz SM. Upstroke wing flexion and the inertial cost of bat flight, Proc. Roy. Soc. B: Biological Sci. 2012; 279, 2945–2950. 10.1098/rspb.2012.0346 PMC3385481 22496186

[31] Bergou A, Vejdani H, Swartz SM, Breuer KS. 2015; Data from: “Falling with Style: Bats Perform Complex Aerial Rotations by Adjusting Wing Inertia”. Dryad Digital Repository. 10.1371/journal.pbio.1002297 PMC4646499 26569116

[32] Featherstone R, Orin D. Robot dynamics: Equations and algorithms, in *Int* *Conf* *Robotics & Automation*. pp. 826–834. San Francisco. IEEE. 2000.

[33] Goldstein H, Poole C, Safko J. Classical Mechanics, Addison-Wesley 3rd edition 2001.

[34] Pesavento U, Wang ZJ. Falling paper: Navier-Stokes solutions, model of fluid forces, and center of mass elevation, Phys. Rev. Lett. 2004; 93, 144501 15524800 10.1103/PhysRevLett.93.144501

[35] Horowitz SS, Cheney CA, Simmons JA. Interaction of vestibular, echolocation, and visual modalities guiding flight by the big brown bat, eptesicus fuscus, J. Vestibular Research 2004; 14, 17–32. 15156093

[36] Geva-Sagiv M, Las L, Yovel Y, Ulanovsky N. Spatial cognition in bats and rats: from sensory acquisition to multiscale maps and navigation, Nature Reviews Neuroscience 2015; 16, 94–108. 10.1038/nrn3888 25601780

[37] Jusufi A, Kawano DT, Libby T, Full RJ. Righting and turning in mid-air using appendage inertia, Bioinsp. Biomim. 2010; 5, 045001 10.1088/1748-3182/5/4/045001 21098954

[38] Vaughan TA. Functional morphology of three bats: *Eumops*, *Myotis*, *Macrotus*, *Univ*. *Kansas Publ*., Mus. Nat. Hist. 1959; 12, 1–153.

[39] Thollesson M, Norberg UM. Moments of inertia of bat wings and body, J. Exp. Bio. 1991; 158, 19–35.

[40] Bahlman JW, Swartz SM, Riskin DK, Breuer KS. Glide performance and aerodynamics of non-equilibrium glides in northern flying squirrels (*Glaucomys sabrinus*), J. Roy. Soc. Interface 2013; 10, 20120794. 23256188 10.1098/rsif.2012.0794 PMC3565731

