# Supplementary figures and images for: Falling with Style: Bats Perform Complex Aerial Rotations by Adjusting Wing Inertia
Source: PLoS Biol. 2015 Nov 16;13(11):e1002297. doi: 10.1371/journal.pbio.1002297 (PMC4646499; doi:10.1371/journal.pbio.1002297)

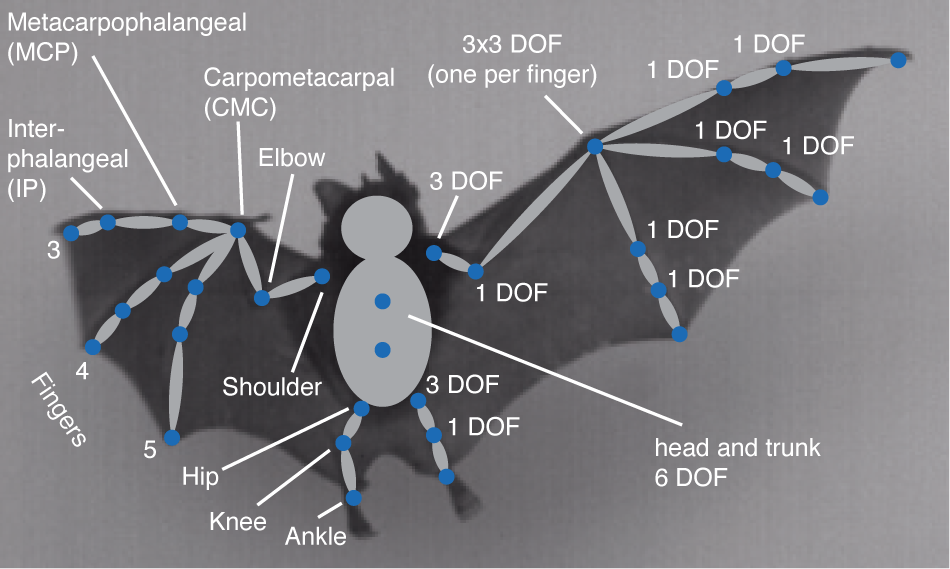

Supplement: S1 Fig — Tracked markers are shown in blue. Each of the modeled joints is labeled on the left side of the figure. The head and trunk of the bat is modeled as a rigid body, and each wing is modeled as 13 rigid bones. Segments are modeled as connected using either ball-and-socket joints with three degrees of freedom, or as uniaxial hinge joints with a single degree of freedom, as indicated on the right side of the figure. The thumb and second finger are not modeled. The carpometacarpal joints are modeled as ball-and-socket joints, one for each of the fingers (3, 4, and 5). Each of the two most distal phalanges of fingers 3, 4, and 5 are modeled as a single bone. (TIF) [file pbio.1002297.s001.tif]

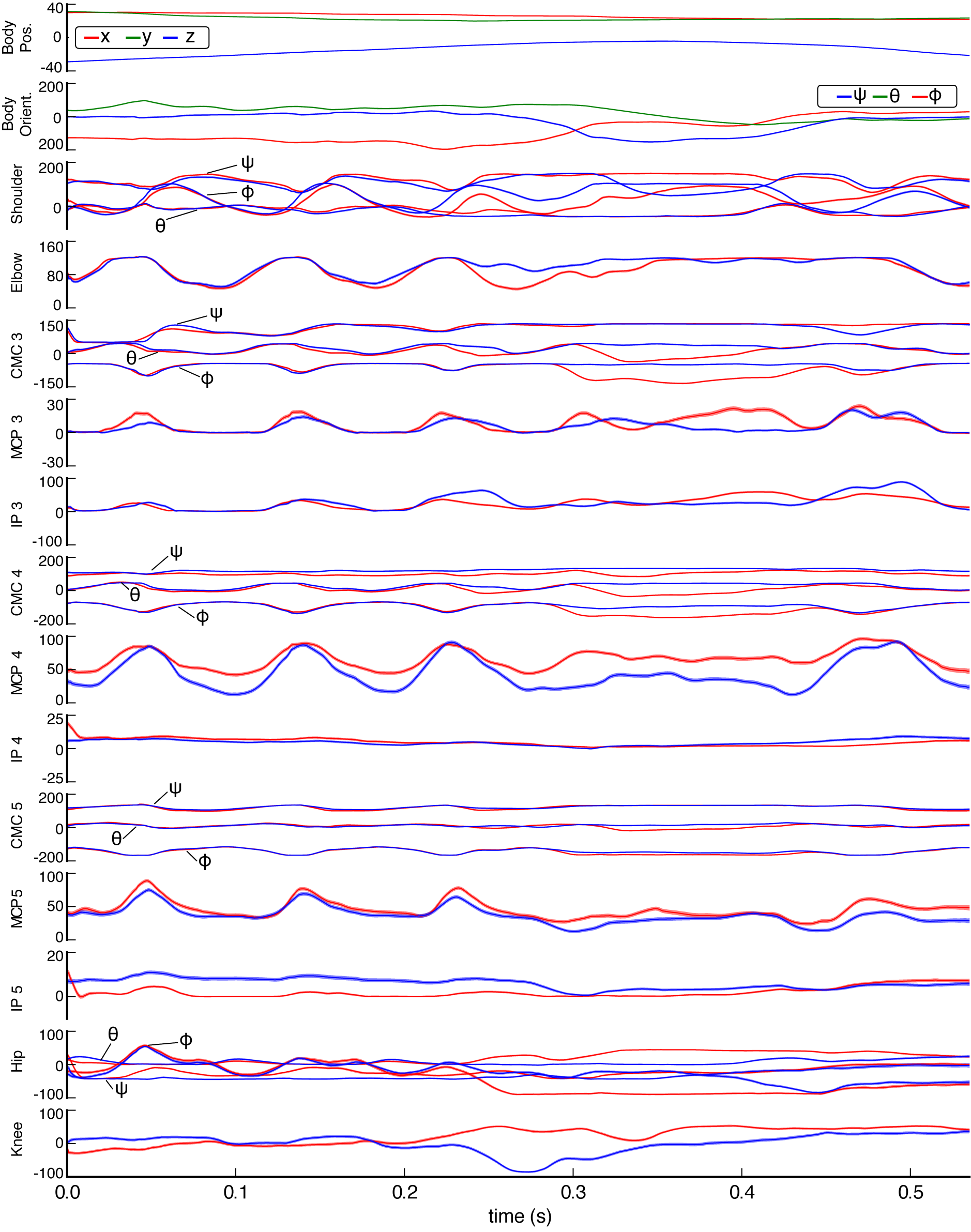

Supplement: S2 Fig — The sequence is shown in Figs 2 and 3 and the joints are as labeled in S1 Fig. Angles are shown in degrees. Kinematic parameters for the right wing are shown in red and the left wing in blue. For multi-axis joints, each degree of freedom is labeled with its corresponding Euler angle. Error bars, estimated by the tracking software, are indicated by the width of the lines. (TIF) [file pbio.1002297.s002.tif]

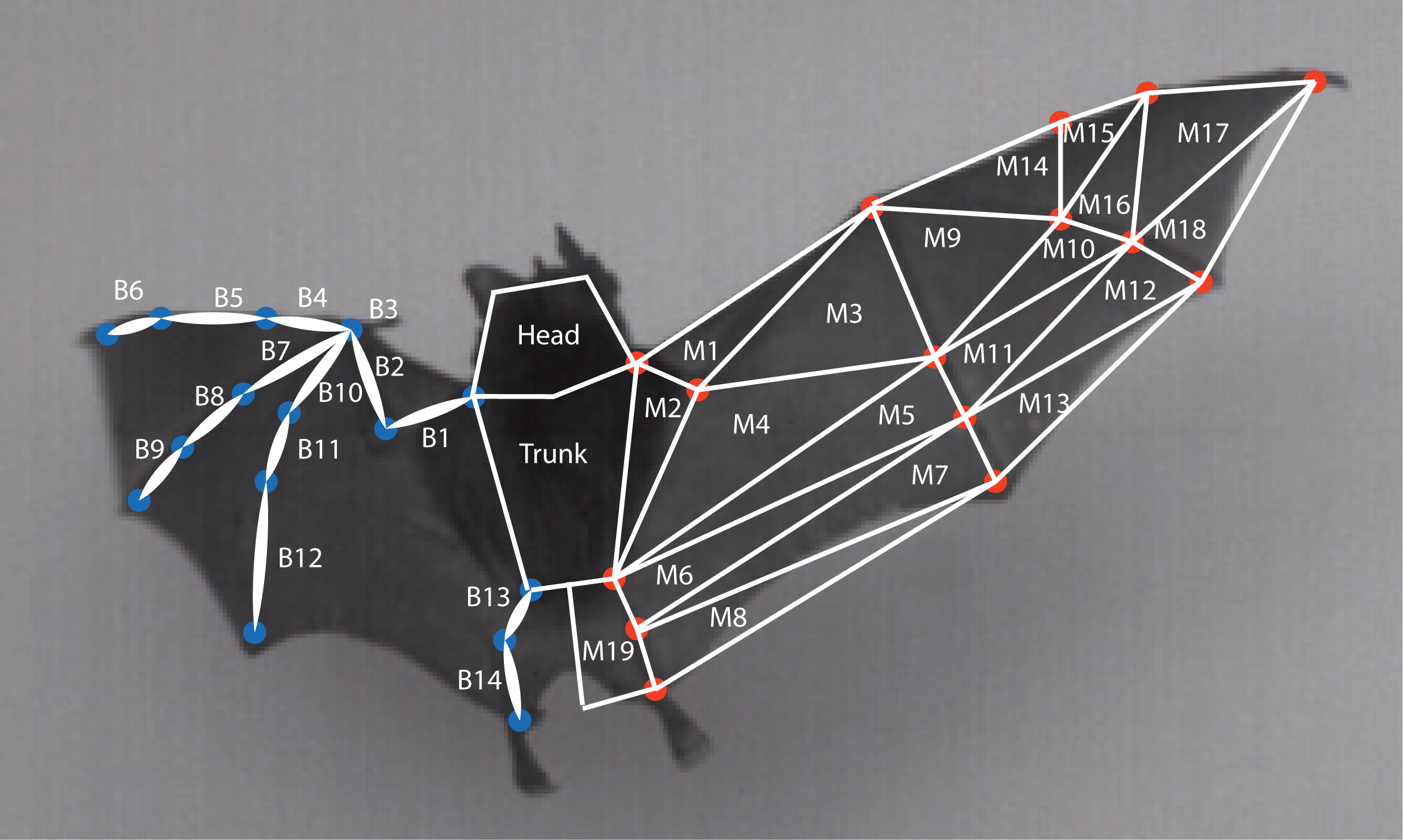

Supplement: S3 Fig — The body was dissected into head and trunk; each wing was divided into 14 bones (B1-B14) and 18 membrane segments (M1–M18), each which were then individually weighed (see S1 and S2 Tables). (TIF) [file pbio.1002297.s003.tif]

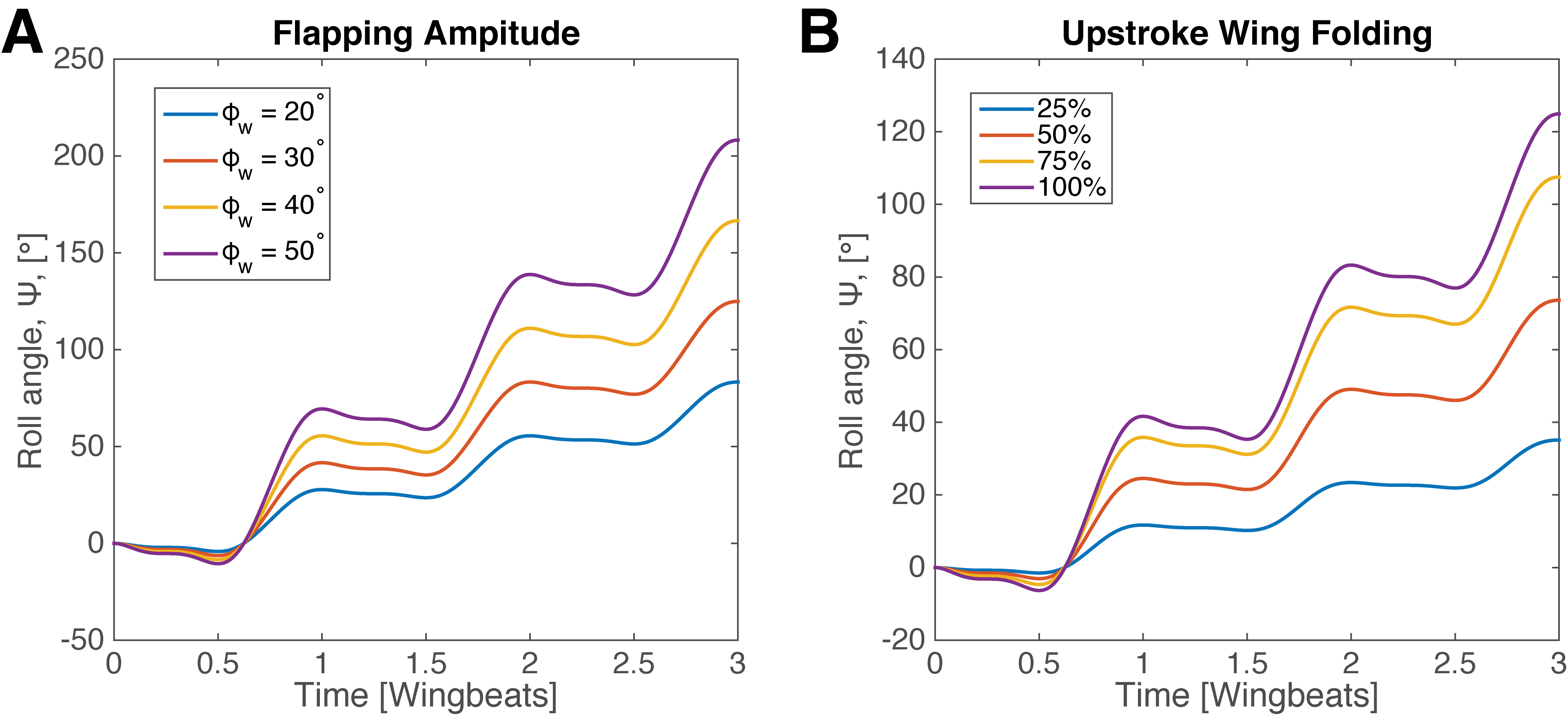

Supplement: S4 Fig — The minimal model is used to simulate changes in roll angle, Ψ, due to asymmetric wing extension. Unless otherwise stated, the kinematic parameter are as defined by Eq 6. A: The flapping amplitude, ϕ w is varied from ±20 to ±50 degrees. B: The degree to which the right wing folds during the upstroke is varied from 25% to 100%. (TIF) [file pbio.1002297.s004.tif]

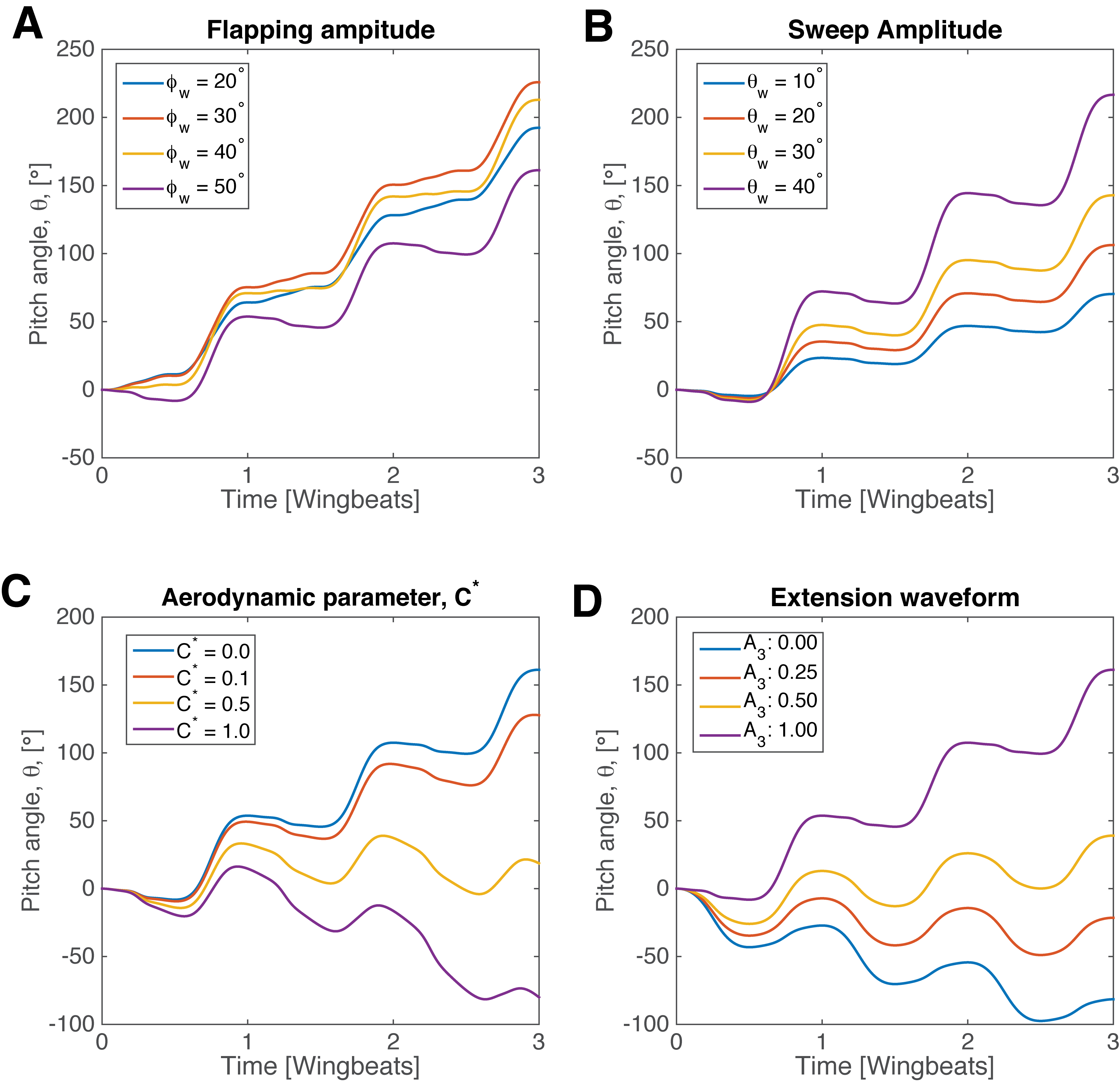

Supplement: S5 Fig — The minimal model is used to simulate changes in pitch angle, θ, due to wing protraction during the upstroke. Unless otherwise stated, the kinematic parameter are as defined by Eq 8. A: The flapping amplitude, ϕ w is varied from ±20 to ±50 degrees. B: The amplitude of the sweep (wing protraction) is varied from 10 to 40 degrees; C: The aerodynamic parameter, C* is varied from 0 to 1; D: The protraction waveform is varied smoothly from pure sinusoidal to the case discussed in Eq 8. This is achieved by re-defining the wing protraction as θl(t)=θr(t)=22.5°(12+2π(sin(2πt)+A33sin(6πt))) and by varying A 3 between 0 and 1. (TIF) [file pbio.1002297.s005.tif]
